# Transcending Measurement: What Matters When Making-with-Music for Equitable Wellbeing in Health and Social Care Systems

**DOI:** 10.3390/bs15091230

**Published:** 2025-09-10

**Authors:** Marisa de Andrade, Pamela Burnard, Deborah McArthur, Aaron Hawthorne, Leah Soweid

**Affiliations:** 1 School of Health in Social Science, University of Edinburgh, Edinburgh EH8 9AG, UK; lsoweid@ed.ac.uk; 2 Faculty of Education, University of Cambridge, Cambridge CB2 8PQ, UK; pab61@cam.ac.uk; 3 North Lanarkshire Council, Motherwell ML1 1PN, UK; mcarthurd@northlan.gov.uk (D.M.); hawthornea@northlan.gov.uk (A.H.)

**Keywords:** music, transcending measurement, health and social care, wellbeing scales, measures, rhizomatic, material-discursive practices, community, health inequalities, systems

## Abstract

Research has long supported the use of and engagement with music as a catalyst for health and wellbeing. However, there is a lack of research exploring how the structures, rituals and ‘minor gestures’ that go alongside music-making, making-with the materiality of music and engagement, can positively impact health. Using assemblages of interconnected community music projects in North Lanarkshire, Scotland, as collective ethnographic entry points, we examine how collective routines and communal activities—through the interplay of material-discursive practices that play out in structural elements, memories, and shared experiences—contribute to the creation of meaningful social exchanges, stability, sense of belonging and becoming. We argue that the benefits of music ‘interventions’ are not solely outcomes from isolated activities, but from the accumulative habits and rituals they affect, offering a new perspective on health as a dynamic process. This reframing invites a transcending of measurement in relation to the impact of music on individual and social wellbeing. Through this, we challenge traditional, conventional wellbeing scales and measures and call for a broader understanding of music’s potential in addressing health inequalities, concluding with implications for scalable community music models that contribute to expanding possibilities for research–practice–policy partnerships in health and social care systems.

## 1. Introduction

Research has long demonstrated the positive impact of music on health and wellbeing. A scoping review of studies on how music affects health and wellbeing by UK healthcare researchers found engaging with music—whether through listening, singing, composing or playing an instrument—positively influences psychosocial variables (Dingle et al., 2021). Specific findings indicated that receptive and intentional music-listening reduce pain (Çetinkaya et al., 2018), enhance social connections (Götell et al., 2009) and improve mood (Fernando et al., 2019). Singing has been shown to decrease agitation and support cognitive health (Mansens et al., 2018), while musical composition has been shown to foster social and cultural inclusion, promoting empowerment among marginalised groups (Bartleet et al., 2016; Travis & Bowman, 2012; Uhlig et al., 2019). 

Much of the existing research focuses on the direct therapeutic effects of music. For example, ‘musicking’ a term coined by music sociologist Christopher Small (Small, 1998), refers to all activities involved in making, experiencing, and relating to music, rather than just the abstract ‘music’ itself. It is a verb encompassing composing, performing, listening and even activities like singing in the shower or taking tickets at a concert. Musicking highlights the social and ritualistic aspects of music, emphasising how it shapes relationships and identities. The meaning of musicking lies in the relationships that are established and enacted through the musical performance. These relationships can be between performers, between performers and audiences and between the individuals and groups engaging with sound as an active process in any setting or community. In a National Institute of Health (NIH) review of ‘the impact of musicking on emotion regulation’, Peters et al. (2024) reported that prevention programmes and public policies should embrace the social, emotional and psychological benefits of music engagement.

Alternative methodological orientations that draw from feminist new materialism and the associated ontological and posthuman turns that carry radical implications for how research is enacted methodologically feature sound-based methods that do not rely on a logic of extraction that essentialises sound as something that pre-exists the research encounter. It is argued that we ‘should seek to unsettle rather than naturalize/neutralize’ understanding sound—through careful engagement—as essential processes from the skin and onto the voice allows the ‘reinscription of the inheritances’ of communities and ‘attending to the relational and affective’ (Shannon & Truman, 2020).

Less attention, however, has been paid to how the structures and rituals surrounding music-making can impact wellbeing. Collective routines and communal activities can contribute to meaningful social exchanges, stability and sense of belonging. Research has established a clear link between sociality, health and wellbeing, with social networks and greater social capital being found to provide a source of resilience (Chatterjee & Noble, 2013; Friedli, 2009). Put simply, participation in community groups is an important determinant of health (Corrigan et al., 2023). Furthermore, Foot and Hopkins (2010) suggest that in addition to having hardships and problems to overcome, our most underserved communities also have social, cultural and material assets that can be identified and mobilised to help them overcome the health challenges they face. More recently, a study (Warran, 2025) highlighted how engagement in the arts—particularly when framed as ‘secular rituals’—serves as powerful interactional routines that foster collective identity, belonging and wellbeing.

While the role of music, making-with-music and music groups as assets are increasingly acknowledged, what matters in measuring its impact remains a challenge. ‘Making-with’ is a term introduced by Donna J. Haraway in her book ‘Staying with the Trouble’, which offers provocative new ways to reconfigure our relations between the human and non-human and the possibility and vitality of ‘making-with’ things. In the context of this paper, we are exploring the power of ‘making-with’ music (Haraway, 2016). Assets here are defined as “cultural, natural and community resources known to improve health outcomes over space and time. They include artists and arts organisations; libraries; museums; heritage sites; green and blue spaces, exercise-related assets; legal or debt advice services and, importantly, the relationships and research-practice-policy partnerships connecting them” (de Andrade et al., 2024).

The fluid, affective and relational dimensions of engaging with music do not fit with conventional metrics, prompting the need for alternative ways of conceptualising and capturing change. Using interconnected music projects in North Lanarkshire, Scotland, as collective ethnographies comprising assemblages, this paper explores the power of shared experiences and the accumulative habits and rituals they affect. The analysis offers a fresh, community-led perspective on health as a dynamic process shaped by a collection of rhythms and relationships.

Collective ethnography is a research approach where multiple researchers—or in our case researchers, practitioners and/or policymakers—collaborate both methodologically and theoretically to study a cultural group or phenomenon, sharing their individual experiences and perspectives to build a more comprehensive understanding (Gordon et al., 2006). Our collective ethnographies—or entangled music projects, scenes or settings—emerge as assemblages that resist unity and favour multiplicity. Working with theory, we analyse the material-discursive practices of making-with-music and how this affects and is affected by those the process encounters. We also trouble the concepts of ‘interventions’ and ‘measurement’ in relation to health and wellbeing in health and social care systems, defining these systems as “constructed mental representations of relationships existing in the world to promote health for people” (de Andrade et al., 2024). We argue that the benefits of music ‘interventions’ are not solely from isolated activities and engagements but also from the accumulative habits and rituals they affect. 

Our analysis of material-discursive practices emphasises the inseparable entanglement of materiality and discourse in shaping reality. This means that material objects (such as drums or other musical instruments), actions (such as drumming) and practices (such as meeting in a community centre to rehearse a musical composition) *are not* simply discursive or determinate representations of assets to help overcome health issues. Indeed, this reductionist framing takes us further away from the affective impact of making-with-music, which is activated by actively participating in its creation and meaning-making thereby, making change visible and possible. 

The significant contribution of this article is, therefore, understanding how benefits of music ‘interventions’ are not solely outcomes from isolated activities but emerge from the accumulative habits and rituals they affect, offering a new perspective on health as a dynamic process. This reframing denotes “transcending measurement” (de Andrade, 2024) or a remaking of self and the ‘thing-power’^1^ of communities making-with-music as a way of expanding health and social care measurement within complex systems. 

## 2. Putting Theory to Work: What Matters?

Our inquiry begins by drawing upon the insights of new materialism and the “horizontal ontology of vibrant matter” (Bennett, 2010, p. 22) to think beyond Western Enlightenment’s binaries of language versus material, body versus mind and measurement versus entanglement. New materialist theorists acknowledge the vibrancy and agency of matter, both human and more-than-human (which include intra-actions with objects, spaces, places, things).^2^ Emphasising the exploration of material-discursive practices^3^ as one of our research approaches, we make an explicit call to the emerging ‘affective turn’ in healthcare research, which values non-cognitive and non-volitional expressions of life, including feelings, animation, tactility and habits (Braidotti, 2013; Roelvink & Zolkos, 2015). From this stance, we focus our attention on the potentiality of ‘sensing bodies^4^ (Cooke et al., 2023), not as containers of inner potential, but as affective intensities always with the capacity to stretch forward—forming relational associations and assemblages that count as ‘currency for social change’ in health and care systems (Bennett, 2010; de Andrade, 2024).

In this paper, we do some rethinking with the ‘vibrant matter’ of ‘making-with’ music by mobilising the central force of ‘thing-power’^5^, and by highlighting the potentiality of the ‘sensing body’, we advance that the ‘measurement market’ needs to ask not just *what* are we measuring, but why are we measuring? We bring assemblages of projects through collective ethnography to illustrate the potentiality of the ‘sensing body’ to be attentive to the emergent; to act in collaboration, cooperation or even in interactive interference with materials and objects that have agency and vitality themselves—‘thing-power’—that enables the mapping of new conditions for ‘transcending measurement’ in health and social care systems.

New materialism brings together a set of post-qualitative theories calling for a shift away from the representational turn, towards the imaginative-material turn, centred on a model of distributive agency across more-than-human entanglements (Truman, 2019). For example, within health and social welfare spaces, this is enacted by a plurality of relationships, where the capacity of things—musical instruments, tables, whiteboards, instruments, tools, worksheets, books, images, pencils, water, props—enters into a mutuality of relations with human bodies; making-with humans and more-than-humans, whereby agency is shared within assemblages (Burnard et al., 2022). Situating our thinking within a broader body of work on the relational potential of music, existing research has looked at how instruments can fill in as ‘active participants’ in both musical and social processes. 

For example, one study (Roda, 2014) considers the skilled tuning of tabla drums and the interactions between the maker, the instrument itself, and the performer. In another (Bates, 2012), the author examines how instruments such the Turkish saz are not merely passive tools but are embedded in the complex relationships with people and practices. Yet another examines how the connections with both human and nonhuman entities (not limited to instruments, but architecture, benches, and objects in shared spaces) shape performance among street performers (Simpson, 2013). These studies further enrich our own observations within this paper. What counts here in terms of the value of ‘measurement’?

Deliberately seeking to dismantle conventional hierarchies of mind over matter and systems of knowledge that do not accurately reflect the lived and felt experiences of community members represented by them, new materialist theories harness new ethical possibilities in health and social care practice settings by reseeing materials as ‘doing’, as active with us, as making-with us in our entanglements. This is the ontological turn that comes by acknowledging what Jane Bennett (Bennett, 2010) describes as a ‘swarm of vitalities’ within our health and social care spaces: “the capacity of things—edibles, commodities, storms, metals … to act as quasi agents or forces with trajectories, propensities, or tendencies of their own … to articulate a vibrant materiality that runs alongside and inside humans”. Bennett calls us to develop new ways of looking and responding, “that enable us to consult nonhumans more closely” (p. 108) and an attuning to the thing-power of matter and matter’s vibrancy.

Significantly, while new materialist theories advance a new ontology of things, this does not mean replacing an old reality with a new one, or one privileged centre for knowing with another. Quite the contrary in fact. Reality is seen as an active and “messy, hyper-complex, high-velocity milieu” (Philippopoulos-Mihalopoulos, 2016, p. 80), already containing eruptive forces of conflict, tensions as well as potentially synergistic attunements:

“… one loses one’s origin, one’s preconceived ideas of location and destination, one’s belief in the importance of the centre. One is lost in a horizontal plane of movement, and on this plane one begins by ebbing and flowing between knowledge and ignorance.”

(Philippopoulos-Mihalopoulos, 2016)

Translated to what counts in ‘transcending measurement’ in health and social care systems, this passage is even more provocative. The convention has it that the world of ‘measurement’ directs attention towards a pre-set goal or when research has an object in mind and a plan with targets set a priori. Rather, from a new materialist stance, we call for a productive coalition of human, non-human and more-than-human bodies that are receptive, responsive and open to the co-constitution of learning, practice, policy and research (Philippopoulos-Mihalopoulos, 2016).

However, this refocusing of attention towards relationships and entanglements with our material partners is not a simple feat. Following Driesch (1914), a reimagining of ourselves within an entanglement of relations and a re-attunement with a more-than-human world will involve a dishabituation. Here is where Bennett (2010) along with Haraway (2016) invite a curious disposition, a playful element in one’s approach and “to be willing to play the fool” (Bennett, 2010, p. 15). This particular stance aims to regenerate highly subjective and individual ways of corresponding with the world and to become perceptually open, obscuring without denying existing tensions and conceptualisations. These correspondences may be productively seen as agential^6^ expressions for their capacity to rearrange previous relations and decide what new forms may become actual. The methodological question is, therefore, left open: how and in what way do such experiences come to matter in our practices? And how might this change the landscape of research and practice in health and social care systems considered to be fixed, rigid and often ‘broken’?

What matters here? After putting theorists to work, we stay with the trouble by repositioning ourselves as practitioner-researchers resounding old research questions in new ways that invite new methodologies and methods that disrupt conventional human exceptionalist systems of thought in order to generate differential powers to reform concepts and practices. ‘Making-with’ often refers to collaborative creative processes and the use of diverse materials in art, design and other creative fields. ‘Making-with’ emphasises the exploration of ideas, experimentation with materials and the development of innovative solutions, often with a focus on engaging with complex situations. It champions open-ended participatory enactment. ‘Making-with’ music involves body–material interferences that are explored, where the combining of materials with bodies are embodied, felt and physically negotiated act as a material–body making-with.

All of this means working differently with data—opening up what counts as data. Here, we enact how the data and researcher are all part of the same world. Here, posthumanism allows us to see how theories, practices and everything is/are always in relation. It follows that we cannot generalise about ways of conducting or knowing things or about groups of people. Resisting the impulse to generalise is a challenge for research because of the substantialising work that language does (Barad, 2007; Braidotti, 2013). The grammar of language brings into existence figurations of the human as a substance with an essence and a self-contained identity. This, in turn, determines how agency, causality and, therefore, ‘measurement’ are conceptualised in health and social care systems and research. 

## 3. Materials and Methodology

Our methodology puts ‘thing-power’ to work across assemblages of interconnected projects, settings or scenes, which in themselves are not empty stages waiting to be furnished with data, but active milieus of bodies, materials and affective relations intra-acting with the force of thing-power. Whilst distinct, these three scenes are not separate.

### 3.1. Situating Collective Ethnography

Ethnographic research has increasingly evolved over the last few decades, moving beyond the lone ethnographer model and welcoming more collaborative and co-produced models—among them being collective ethnography. In this paper, we use collective ethnography to describe a multi-site/researcher approach where multiple entangled music projects function as collective ‘entry-points’ into a shared inquiry. 

Collective ethnography shares links and overlaps with other forms of ethnography such as collaborative ethnography (Lassiter, 2005, 2019), duoethnography (Sawyer & Norris, 2012), and team ethnography (Erickson & Stull, 1998). All stress the importance of multi-researcher input and collaboration and the value of sharing experiences, reflections, and interpretations to build a more comprehensive understanding. Rather than producing a single narrative or perspective, collective ethnography is composed of various stories (Gordon et al., 2006).

Within the academic literature, collective ethnography has been applied a number of times in various settings. In a collective ethnography spanning three decades, a group of researchers engaged in fieldwork on a sugarcane plantation in Brazil, using this collaborative research method to form a nuanced and comprehensive portrayal of the socio-economic landscape and labour rights, capturing the complexities of social change (Sigaud, 2008). Meanwhile, an Academic Medicine research report applied collective ethnography to explore how attending physicians at select medical training units manage the complexities of their roles as both doctors and teachers, using the observations to generate themes to identify important next steps (Lemaire et al., 2017). In another paper, the concept of remoteness during the COVID-19 pandemic was captured through a collective ethnography carried out by a multitude of researchers showing it as a complex emotional, social and political experience rather than just a geographic distance (Martínez et al., 2021). 

The latter relates most closely with our inquiry. We seek to show how North Lanarkshire community groups gathering *through* music benefit not only from the music itself, but from the complex, entangled, relational encounters opened up through music-making, where meaning and measurement are co-created through the very act of participation. The collective, multi-site aspects of our approach are particularly effective in gaining access to community members’ lived experience and contribution to the cyclical evaluation process.

### 3.2. The Interconnected Projects and Practices in Our Assemblages

Our assemblages are situated in North Lanarkshire, Scotland’s fourth-largest local authority with a resident population of approximately 340,000 facing unique health inequities. It has the highest rate of school exclusions among children in care, with 24.8% of children living in poverty compared to the national average of 23%. Additionally, over 21,000 residents live in the 5% most-deprived areas, with 75,000 within the poorest 15% data zones (North Lanarkshire Council, 2019). To tackle this, North Lanarkshire Council approved an innovative five-year plan for arts and creative community activities in the local authority area in 2024 in collaboration with various multi-sectoral partners cutting across health, social care, justice, education and employability. The basis for this regional strategy is ongoing transdisciplinary applied research funding addressing health inequalities through the arts led by one of the authors (de Andrade), with a second author conducting applied fieldwork in North Lanarkshire as its community-embedded researcher (McArthur) and a third managing the overarching research consortium (Soweid).

Significant learnings from our consortium called REALITIES in Health Disparities—which stands for Researching Evidence-based Alternatives in Living, Imaginative, Traumatised, Integrated, Embodied Systems—is evidencing the value of creative engagement as more-than metrics or key performance indicators for community wellbeing. We recognise that being well is a dynamic, affective state of being and doing that can be valued through mediums and models that are not transactional, unlike the various existing health and social care systems our consortium continuously encounters. 

For the purposes of this paper, we focus on one asset hub (North Lanarkshire) out of five (our consortium extends across Clackmannanshire, Dundee, Edinburgh and Easter Ross in the Scottish Highlands) and one arts-informed approach namely music-making (we engage with multiple other creative as well as qualitative and quantitative approaches in REALITIES (de Andrade et al., 2024). In collaboration with transdisciplinary academics, practice partners (such as schools and Health Improvement) and local communities, North Lanarkshire Council’s Arts Development Team has co-produced a series of creative engagements, which includes All Ability Music and the North Lanarkshire Community Choir, Ah Cannae Sing [I Cannot Sing] and Sensory Storytelling to help tackle inequity and improve wellbeing.

What binds these four different groups together is not just their creative form (music), but their focus on relational modes of engagement—relationship building, experience sharing, and wellbeing support. These modes of engagement pull away from standard models of ‘intervention’; together, they form practice assemblages—that is, flexible, ever-evolving configurations of people, settings, places, emotions, and materials that blend together to create meaningful, visible change over time. Rather than taking each of these projects as individual entities, we treat them as parts of a whole that responds to need, connects communities, and offers different ways to understanding health and wellbeing.

### 3.3. Data Collection, Meaning-Making and Making-with-Music in Our Assemblages

At this stage of the inquiry, our paper—following the dominant paradigm in Behaviour Sciences—would be expected to describe the data collection and analytical process with sufficient details to allow others to replicate and build on the published results. By now, it should be clear, however, that presenting and interpreting empirical data in this way—to generate actionable, instrumentalised, generalisable results for repeated application—would be counter to our core argument. Stuck between a (methodological) rock and a hard place, we are compelled here to explain our method through a linear, ‘traditional’ delineation of data collection (see Table 1), while acknowledging that our projects cut across time, space and matter in different ways due to the very nature of our post-qualitative inquiry.

The data from the music groups was collected by McArthur and Hawthorne, both members of North Lanarkshire Council. This data was originally gathered to evaluate and review the music programmes and workshops within the scope of the Council’s Arts Development arm between 2023 and 2025. For this article, this existing local authority data and feedback has been repurposed to explore the broader question regarding the benefits participants experienced from being part of a music group. 

Feedback included surveys, interviews, project evaluation reports and podcasts that were recorded with community members taking part in sessions. Verbatim transcripts of these iterative, unstructured interviews were then used for evaluation purposes to reflect on changes in perceptions and improvements in health and social wellbeing. In some cases, carers needed to complete feedback on behalf of participants, so rigour, interpretation and honesty was reliant on them. Feedback was collected at least once an academic year, with regular project feedback loops to ensure facilitators and the Council were reflective throughout. Data was collected at baseline once the project commenced, at the mid-point and upon completion of delivered sessions. 

Two of us are community arts practitioners (McArthur and Hawthorne) advocating wellbeing through creative engagement and connected to informal health and social care systems through local government (North Lanarkshire Council). They worked directly in the field with the community members and were core members of the team delivering the music sessions along with local facilitators. Their close involvement gave them first-hand access to all of the relevant information. Two of us are academics with extensive experience in arts, creativities and educations (Burnard) and creative health, science and society with a focus on inequalities in health and social care systems (de Andrade). One of us is an academic project manager with research, practice and policy experience (Soweid). Collectively, we are (at least) the human elements of these assemblages along with all the community members we encountered along the way, either through in-person music engagements or through text, audiovisual or other forms of sensory ‘data’ in the meaning-making process. All authors worked together in their attempt to translate post-qualitative theory into accessible practice-based key learning questions. We interviewed each other on Teams throughout the analytical process, recording and transcribing our contributions.

As part of the ‘multiplicities’ (Deleuze et al., 2013) within our practice assemblages, we also bring social, material and technical components that move with us as we make-with music as non-measurement (more on this in the Section 4). Together, we have engaged with these projects—directly or indirectly, humanly or virtually, theoretically or practically—multiple times during the lifespans of these engagements, and also long before they were conceptualised and enacted in North Lanarkshire and beyond. We have been thinking with these projects (and many others like them) during the duration of this study and will continue to think with these concepts long after this study ends. The work is enacted across an interplay of material-discursive practices that play out through ‘mind-body-soul memories’ and in structural elements (sound, harmony, melody, rhythm), memories, and shared experiences—contributing to the creation of meaningful social exchanges, stability, sense of belonging and becoming. 

Ethical approval was received from the University of Edinburgh’s School of Health in Social Science through institutional processes and protocols. As a consortium, REALITIES also promotes relational ethics and critiques accepted definitions of vulnerability and risk in ethics processes that do not seem to be aligned with the wants and needs of the underserved communities we are researching with (Jundler et al., forthcoming).

In this article, we apply Karen Barad’s concept of ‘ethico-onto-epistemology’ (Barad, 2007) to ethical caring and response-ability in this research, where we are continually inspired by the locations of possibility opened by Barad’s thinking and wondering. We also consider Barad’s emphasis on ethical ‘response-ability’ and ‘intra-actions’ (Barad, 2007) as a profound commitment to ethical caring and the wellbeing of others and the environment, as well as an insistence on caring about the application of an ethics of care in research—an urgent matter. We address the impact of ethical caring on the researcher’s positionality and the imperative of transparent research practices that embody care and demonstrate care in practice. 

As we write this article, we know that we are struggling to grapple with the ethical entanglements we currently face as researchers, and yet it is from our experiences within the tradition of social science research that we can and must respond to the question, “What sort of world do we want to live in?” More questions begin to unfold in the search for a response. How can we take responsibility for the ethical dimensions of production, experience and transformation in research? How do relationalities involve us in seeing ourselves as part of the world, appreciating the intertwining of ethics, knowing, and being as researchers? And when we are asked, quite simply and plainly, “Got ethics?,” what words do we use to frame this world in which we live and work?

## 4. Results: Mattering Measurement

In this section, we turn our attention to the findings that emerged as we explored what matters in measurement. Inspired by Manning (2013), our research positions itself as a “a minor gesture”—“a force that challenges received wisdom and common sense (the ‘major’) by offering potentially unlimited experiential variations that suggest alternative forms of being, knowing and doing” (Simmons, 2018). 

The ‘major’, in this paper, is the ‘measurement market’ that privileges ‘validated’, ‘credible’, ‘practically tested’ measures, frameworks and approaches that claim to demonstrate “value for money and trustworthiness”—rather than those that are more relational, dynamic, embodied and connected to an individual or community’s ever-evolving lived and felt experiences (de Andrade, 2024). Wellbeing scales are included in this ‘major’, alongside Key Performance Indicator (KPI). metrics, targets, indicators and outcomes that are fixed without centring the person or population meant to be benefiting. 

**Figure 1:**
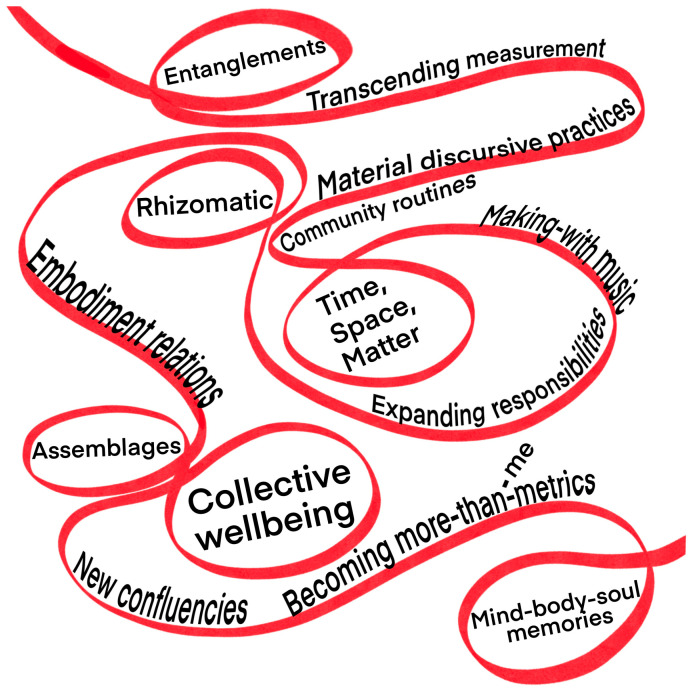
A mapping cartography of our entangled multi-site assemblages reflecting key findings.

Our analysis unfolds by focusing on the conditions of emergence for ‘non-measurement’ in our assemblages of making-with-music, such as collective routines and communal activities and also structural elements (sound, harmony, melody, rhythm), memories and shared experiences in our projects and practices. Firstly, we reflect on rituals and objects as mind–body–soul memories in non-measurement. We then work with findings from our assemblages to frame time, space and matter as entangled systems of non-measurement. This is followed by illustrations of meaningful social exchanges and stability as musical non-measurement. Finally, we reveal how something new emerged in these systems and practices—namely, assemblages *becoming* more-than metrics in non-measurement. Figure 1 provides a visual illustration of the entanglements of our multi-site assemblages.

### 4.1. Rituals and Objects as Mind–Body–Soul Memories in Non-Measurement

“When I’m thinking about the sensory storytelling project, it’s the rituals and the habits for these young people with additional support needs and complex needs. Some of them have no language, but those habits and rituals is what connected to memory, and that then meant that they started to know when a piece of music began, what was happening. You know, it’s those cues of understanding what’s coming next…”

Deborah’s (author 3) voice drifted as she searched her mind’s eye for the right words to capture that moment in that classroom surrounded by whiteboards and a storybook of “The Snail and the Whale”: “When in a traditional story setting, they might not be able to follow the music… it’s the cues that you add within the workshop… that’s what’s then making that connection into the body, into the soul. You know, you could see them start to smile when music came on” (interview).

Rather than simply reading the book from start to finish, the idea was to bring each ‘thing’ in the book to life and connect to it through embodied sense-making. If there was mud, the musical storytellers would have trays of mud so the young people could touch it. If there was water, they would have a bucket of water or water spray. Using material objects as literacy prompts, the ‘thing-power’ of the mud or water resonated at the start of the song-story, again in the middle as the tale unfolded, and at the end when the narrative quieted. Storytelling and music-making lifted the story off the page and into the minds-bodies-souls of the young people, who were raised by creating an “original little song” inspired by a fictional, restless snail on its travelling whale companion. 

The introductory piece of music was used to move the story along and also sung at the end; punctuating the session while acting as an invisible calibrator throughout the thirty-minute session: “And from our process and the way that we worked, it was about drawing out themes and feelings and going beyond the world in which the book is set…so if we’re in a pool of water, we’re really exploring what that water is, what’s in it, what does it feel, what does it smell like? What does it touch up against? That was really important for the storytelling, because some of these young people’s engagement can’t be through speaking or it can’t be through touch because they’re adverse to being touched or touching things. We really had to think of that multisensory element for everything that we did” (Deborah, interview).

An opera singer, musicians playing brass guitar and the fiddle, singers and storytellers worked in pairs, so young people did not experience everybody—or too much of the ‘thing-power’—at the one time. That would be too big, too much for some with additional support needs (ASN) to cope with. But instead of being overwhelmed, the same song would start every session, and an image would be held up to the class, representing their story. The same song and same image, telling a sensory story over six weeks:

“And what was so beautiful about it was the rituals that we created. The way that we rolled in our trolley every week and the trolley had all of the materials on it. And so that trolley was a visual representation. Then the music was like a sound representation. The musicians also walked round the space the same way at the start of every session, and the young people could come and feel the vibrations on the instruments. And so, if they weren’t connected, they could connect with the vibration because it was the same piece of music at the start. The vibration would be similar, so it was just that way of connecting in”.

(Deborah, interview)

School staff soon reported that when young people heard the song, some of them would want teachers to know that the song was something material, like a plant—a ‘thing’ that they could express their excitement through. They would run to the image and point. An indiscernible connective tissue emerged relating the music, to the story and to the artists:

“They know who the trolley is, they know what day of the week it is because we went in on the same day of the week as well. And so, the teacher would be able to say ‘it’s Thursday and what happens on a Thursday?’ then they played the song. And so, as these connections were made, then the young people were able to evaluate [or measure the value of the project] using board maker symbols and a word that our ASN schools use. They were able to lay lots of board maker symbols out in front of them about: ‘How did it make them feel? Did they enjoy it? What did they get out of it?’ And they were able to point to different symbols”.

(Deborah, interview)

The music became a marker, measure or indicator for the school to use as an act of literacy as, according to their age and stage level and ability, these young people were in fact able to follow a book from beginning to end and understand the characters and the themes and also how it connected to their emotional wellbeing and how it made them feel—all through the medium of music as non-measurement:

“Through weekly observations of pupil responses, interactions, engagement, feelings/emotions and expressions, it was evident that the project was a very positive experience for our pupils. Stevie learned to say ‘whale’, so she would say this on arrival to class on Thursday. We displayed art work that they did during one session; this was a reminder of the story project to look back on, and the children would often use the word ‘snail’ and ‘whale’ whilst pointing to the pictures. We added a snail and a whale toy to our stuffed toy tray and the pupils would explore the story though props on a daily basis. When asked about their favourite bit of the story project, the children offered a word or a gesture. Stevie gestured a trumpet—she really enjoys music and musical instruments. She was given the opportunity to see and hear new instruments. Graeme said the word ‘jellyfish’—he enjoyed holding up the umbrella prop that acted as a jellyfish. Riley said ‘snails’—she really enjoyed exploring the different coloured snails”.

(interview, teacher)

It is poignant to reflect that in the ‘real world’ rather than this ‘magical reality’ co-created with musicians, teachers and young people, snails also hear by sensing vibrations through their bodies. These non-humans feel their environment through touch; their forms rubbing up against other ‘assemblages’ to detect sensations and make sense of materials around them. Perhaps, much like humans in touch with the more-than-human, they are also in touch with these moments of ‘magical realism’: “When you are relating so deeply that you feel alive, awake. When you experience reality as something different. When you feel much lighter, clearer. Lucid. When you experience an alternative perception of time” (de Andrade, 2022, p. 35).

### 4.2. Time, Space and Matter as Entangled Systems of Non-Measurement

In Sensory Storytelling, musicians and facilitators became skilled at abandoning linear time, for example, by forcing sessions to be an hour long when they sensed students were becoming unsettled or over-stimulated. When the experience of staying-with the music-making became too much for some young people, instrumentalists would inherently know when to wrap up. In these moments, musicians replaced the melody for props—consumable materials brought along to sessions for young people to chew on or “really get into them”. As Deborah explained

“We weren’t going to ask for them [objects] back and take them to the next class, but the nature of those types of materials meant that the young people could keep playing at their own engagement and level, but the session could end and they could jump off their trumpet as well… [the young people kept] playing with the prop or they could go and sit in their corner or they could go and walk around their classroom, but they were still engaging. One of the most engaging was a tiny little snail that we had and you could pull it back and it would race off, but they would run them over their bodies and they would, they would run them up and down their hand and they would want to chew them and bite them. But one little boy was keeping them safe in his pocket, so he’d really connected with this little snail… and I think that’s it. You can never understand the way in which people are going to connect with the material that you offer, but you just have to have multiple layers of engagement and multiple points for them to connect with and then whichever one that is for them… it can’t be prescriptive”.

(interview)

We are struck by complementary contradictions in these assemblages—time being expansive and shortened; vibrations building excitement being ‘pulled back’ before the point of sensed over-stimulation; the runaway “tiny little snail” moving fast over bodies and then slow. The rhythmic pace of the sessions—fast, slow, compression, decompression—the vibrational energy of it all as entangled time-space-matter or “spacetimemattering”(Barad, 2007)^7^: making-with-music as a transcending of measurement. As Deborah reflected

“… in any session, there is always a rhythm… it ‘sits in you’. You know when it’s in time and you know when it’s off because you can feel it, you can feel it in the people. You can feel it in the response. You can see it. You know it’s something quite palpable sometimes when you’re off beat with your group or with the piece… when you’re really trying to find that rhythm. And then when you find it, I always feel like my insides go ‘and here we are’. You know that way? Like now we can dance together, you know… I don’t know how you write that, but it is a feeling. It is a feeling”.

(interview)

This affective energy sensed by all in the space motivated facilitators, musicians and students alike—“the whole group” to “be on their feet helping… to get really up close”:

“I’m just thinking about Ryan [a musician] playing a saxophone that a little boy had his arms just linked right round it and Ryan was in the weirdest position trying to make this happen, but he just wanted to feel the vibration of it. And then Ryan had a noise for the wheel and one other little boy wanted to know how to make it. So, Ryan allowed him to put his fingers on the keys and he thought he was making that wheel sound, so to see his face of that engagement was really beautiful…”.

(Deborah, interview)

As Aaron (author 4) noted, all of this was unfolding within a space—not “a big reverberant hall” with a “huge resonating instrument almost sort of blowing the roof tiles off”, but a classroom that changed the “dynamics completely”. Teachers noted how the “quite low ceilings” and “carpeted” environment made for a “totally different experience” with those “frequencies reacting in the room with the things in the room”, which was “amazing”. The “flipped dynamic” and “sense of ownership of their space”—interconnected with attendees’ non-linear experiences of time and engagement with material-discursive practices—contributed to the creation of meaningful social exchanges, stability and becoming (interview).

### 4.3. Meaningful Social Exchanges and Stability as Musical Non-Measurement

Across all projects, participants were vocal about the benefits and joy that came with the social aspect of being part of a music group. The settings provided opportunities for material-discursive practices, where emotional exchanges and social connections were both shaped by and expressed through music-making. In one example, an art tutor for the All Ability Music group noted that for people with additional support needs, classes created the environment to foster friendships that they otherwise would not have been able to:

“… they are quite isolated because they’re just with their carers, so it gives them a wee bit of autonomy. Even deciding what chair to sit on, that’s a big deal. And then making friends within the group, maybe someone decides they want to sing a song and do a duet with someone else in the group—you don’t get that chance because if they have to be with friends, they always have to have a carer with them”.

(interview)

Meanwhile, North Lanarkshire choir members emphasised the importance of social support through community making-with-music to improve their health outcomes. Nadine noted: “For me, continuing with the choir has not only allowed me to continue singing, but has also introduced me to a new group of friends. I recently went through a health hiccup and everyone at the choir helped and supported me whenever I could attend. I’m sure this improved my recovery” (questionnaire). Annie, a teacher for a class of children in the Sensory Storytelling project, added: “The class interacted with the staff and became familiar with them over each session. This had a positive impact on pupil confidence, acceptance or unfamiliar adults visiting our class and pupil engagement and participation in new experiences” (questionnaire).

The materiality of instruments, chairs and props were imbued with social meaning—even the songs themselves became a medium through which participants engaged in friendship and social interaction. Such exchanges highlight how making-with-music is a relational practice: a space where individuals build community and experience recognition. The social connections are not just in the music itself—they are enacted through it and with it. As Aaron stated: “It’s what the arts does best—it got people doing the thing, but it also created a wonderful safe space for people to get talking” (interview).

Drawing from quotes and feedback from the music programmes, there were echoes of how the groups function as an anchor—something familiar—in their weekly rhythms. The reliability of the groups, from the repetition of music sessions to the structures they follow, provided a material-discursive container of routine—be there at the same time of week, same location, and/or same attendees/instructors. This contributed to the environments being transformed into something secure. One All Ability Music attendee, Sandra, commented: “I like meeting the people that run the class… I love chatting with you every week, I come in and you’re asking how I am.” Additionally, her support worker, Monica, shared that she had noticed Sandra becoming more tolerant and sociable since starting the class: “I’ve noticed a massive difference in her” (questionnaire). Other support workers noted a distinct change in an individual if the routine changed: “I would say out of all the activities we do, if this class isn’t on (school holiday-wise), it does really affect him, and he will always ask for it. If the class is cancelled, then he’s really upset” (questionnaire). 

Regular, embodied participation across the ongoing making-with-music groups has become integral to how people experience their weeks, also serving as a marker for loss. As Katie, a community choir member reflected “Without the joy, release, and escapism of singing, I think I would feel a massive void in my life… I feel singing is vitally important to my sense of wellbeing. When I don’t get to sing, I miss it very much. I enjoy the company too and have developed very close and, I believe, lifelong friendships with some wonderful people” (questionnaire). 

The stability surrounding these friendship circles was also emphasised by facilitators, as “repetition was really, really important”: “so the time they [community members] get the bus. They get the same bus every week. It’s the same bus driver that you see, because that’s that person’s route and schedule, as well as the same café, at the same time. What you’re eating when you come to the class… even just a change of time whether it’s, you know, ‘we’re changing half an hour, we’re changing it a day’, then the ripple effect. Also, the safety they feel in that” (Deborah, interview).

So many little connections that are made for someone to feel safe, find enjoyment and be well—room layout, lighting, temperature, the playlist for the day. All of these little intra-actions and ‘things’ that connect for someone to really receive the energy in the room with others. 

### 4.4. Becoming More-Than ‘Me’ and More-Than Metrics in Non-Measurement

By staying close to these meaningful social exchanges in the space and tracing their relational happenings (Bondi & Fewell, 2016) something new emerged from these musical engagements between choir members, school facilitators, music instructors and community members. As well as being an “avenue” for friendships and continuing friendships” or a catalyst for “that feel-good factor that you don’t want to miss each week” (Chrissy, interview), communal music-making opened up an unlimited space for perpetual movement or ‘becoming’.

Individuals arrived as ‘me’ and left as ‘we’, individually and mutually transformed in some way through participation in the projects. The relational, material-discursive practices shifted attendees from a ‘self’ into part of a whole—becoming a collection of ‘selves’—emergence of a fluid, entangled, relational force that is, in itself, a non-measure of wellbeing. The evidence of this in relation to improved health outcomes came later, also through relational encounters when facilitators would bump into the choir or other community members at events. It was then that the mental and physical health advantages of making-with-music in these mind–body–soul, time-space-matter entanglements were verbalised by participants, who were going to their doctors to overcome specific conditions. 

They would frequently report how, for example, after four weeks of coming into music sessions and performing “breathing every week and singing”, they would get better: “the transformation from when you [a participant] walks through the door and begins the class to when they’re going out there is a complete transformation in a person’s at least physical appearance. You know how loose they are, how there’s usually a smile. You know, there’s just a difference… again talking about rhythm thing. You’re leaving the room, going out into the world with a much different rhythm going than you did when you came into it” (Aaron, interview).

What counts here in terms of the value of ‘measurement’ is the quality of relationships within these assemblages, where musicians and facilitators are not disconnected performers learning their music, set lists and shows. Instead, they are embodied, responsive, attuned individuals, who are part of a whole, working collaboratively to promote a healthy system. As is the case in complex systems, this entails

“… complete uncertainty when you’re delivering a workshop, because it’s not you standing up presenting to someone. There is an interaction and a push and pull, and a give and take… there’s going to be someone trying to get at your instrument or wanting the prop that you’ve got or wanting to take your hand because they really want to engage you and another element of the exercise. And there you are playing your fiddle. I’m thinking of one of our musicians. She’s holding her fiddle, but they’ve got her by the elbow and they’re trying to bring it over, you know, 360… I don’t think you can ever be 100% prepared and that brings me back to that rhythm and feeling. It’s like you’re there. You’re improvising with the people in the room on a holistic level, where you’re feeling from them, so you’re giving them something from the feeling that you’re getting from them and it’s that back and forth… you are there to go with the flow and follow the story and respond to what the young people give. And when they have had enough music, [have] pieces [props] in your bag that you can pull out at any moment to keep the session going…”.

(Deborah, interview)

Deborah moves her hand like a conductor, her body sways from side to side, her voice drifts off. Once again, she searches her mind’s eye for the right words to capture this moment. I (Marisa) find myself moving from side to side too, our bodies mirroring each other on screen in a virtual space.

“I don’t know how you write about that, Marisa,” she says without realising she is writing the script herself. “I’m sure you will do it beautifully, but I keep going like this with it… [hands like conductor, bodies like branches on a tree in the wind]… because that’s what it is. How do you turn that into something that you can write? It is a push and pull and a dance. It’s really holistic feeling.”

Marisa thinks to herself I’m becoming more-than ‘me’. I am more than metrics. 

## 5. Discussion: The Value of Non-Measurement in Healthy Systems

Despite well-intentioned efforts in health and social care—not least sector integration mandated by the Public Bodies Joint Working (Scotland) Act 2014 more than a decade ago to promote healthy communities and tackle inequalities—researchers and practitioners are still navigating complex, fragmented systems within this policy landscape. While ‘the system’ is criticised for being broken and traumatised, visionaries and executors are attempting radical transformation and whole-system redesign at local and regional levels (through efforts like the ones put forward in this paper connecting the arts to health and social care and education amongst other sectors); national levels (for example, through the mobilising community assets programme, which REALITIES is a part of); and globally (such as the Human Learning Systems approach to public service) (Centre for Public Impact, 2025; de Andrade et al., 2024).

Fundamental to this paradigm shift is challenging common assumptions about the value of ‘measurement’ as systems set out to serve the society of the powerful, in order to bring forth the power of a different gesture: one that nudges our attention away from what matters in terms of being well. This reframing denotes a transcending of measurement (de Andrade, 2024) in relation to the impact of the creative-relational, such as music-making, on individual and social wellbeing. 

It is clear from our work that traditional, conventional wellbeing scales and measures fall short when it comes to evidencing the lived and felt experiences of people with complex needs and wants in complex systems. Using extractive and reductionist approaches to measure these individuals’ or groups’ wellbeing may lead to “violent evaluation” rather than measurement scales that are “peaceful, playful, ecological and rational” (Roberts, 2025) and indeed helpful. Our research draws attention to the importance of collective routines and communal activities—through the interplay of material-discursive practices that play out in structural elements (sound, harmony, melody, rhythm), memories, and shared experiences—to contribute to the creation of meaningful social exchanges, stability and a sense of belonging and becoming. This opens up possibilities for research–practice–policy partnerships in health and social care systems to embrace, apply and mainstream participatory and affective non-measurement and evaluation design to confront bias, power, and the illusion of objectivity (McLean et al., 2025). 

## 6. Transcending Measurement Matters

Upon tracing the rhythmic, relational practices of community making-with-music, this paper has highlighted how wellbeing cannot be simplified through individualist measures and scales; rather, it is an emergent, dynamic, collective force generated through the material, affective entanglements and relationships of everyday happenings. By being entwined in mind–body–soul collectives of community members, facilitators, carers, staff, researchers, practitioners, policymakers and musicians, we have shown how creative, embodied, day-to-day routines are so much more than entertainment or hobbies—they actively contribute to a sense of safety and self, familiarity and shared identity. 

Taking part in these musical groups creates spaces for growing connections, as well as temporal anchors in the weekly lives of participants. Most importantly, the stories and exchanges scattered throughout our engagements challenge linear metrics and measurements. As wellbeing is invited through gestures, sounds and the comfortable predictability of chairs, instruments, movements and people—the ordinary everyday—benefits cannot be captured through traditional evaluation models and tools. Our research, therefore, contributes to a growing movement of reimagining health and social care systems through creative-relational processes. 

In order for policy and practice to evolve health and social care into systems that truly serve communities, promote wellbeing and address inequalities, we must continue to think outside the box of how value and impact can be understood and reimagined. Policymakers are encouraged to embrace the potential of the affective and material-discursive engagements as essential ‘community assets’ that support health and wellbeing in complex systems. We must think and move beyond conventional and reductionist measurement approaches; a paradigm shift away from linear, outcome-driven frameworks is needed, towards approaches which foreground relational and embodied dimensions and wellbeing. This, in turn, will push against existing power dynamics and biases across measurements and metrics, contributing to whole-system(s) redesign. Moving forward, policies—and their evaluations—must nurture the multiple, interconnected, dynamic realities of those experiencing poor health or inequalities.

## 7. Limitations, Future Research and Key Learning Questions

But what does all of this conceptual and theoretical thinking mean in practical terms for practice-led evaluation and policymaking in complex health and social care systems? While our overarching point about transcending the measurement paradigm may be very welcome—if not visionary—questions do not go away about how one evaluates, improves and adapts this kind of work, as well as the pragmatic questions of how one convinces an external audience with multiple stakeholders and transdisciplinary academics of what it affords. Drawing from feedback from questionnaires and other traditional qualitative and quantitative evaluation approaches helps us move towards a more holistic approach to integrating lived and felt experience into normative evaluations, but to be really effective and convincing, we need to reflect on *how* this kind of work might fit into a world where the measurement paradigm dominates and, indeed, how the two approaches are complementary. We acknowledge the difficulty in capturing the depth of the music programmes through conventional evidence-based tools and propose that evaluation should blend qualitative, relational insights with more formal measures such as questionnaires and surveys to ensure the strengths and limitations of different methodologies are balanced.

So-called expert, academic, objective evaluators need to be de-centred to make space for co-producing knowledge about improvements in health and wellbeing with community and practice-led assessors. This is not a straightforward exercise, as it throws up questions of bias, potential power imbalances, conflicts of interest and uncertainty about whether data is being ‘cherrypicked’ to support a particular position or paid for service. Transparency is key.

Furthermore, part of the feedback we receive about applying post-qualitative research in practice and policy settings is how inaccessible it can be for readers, thinkers and especially practitioners engaging with these concepts for the first time. We accept this as a current limitation of our theoretically dense contribution, though we offer this research as the next step on the applied post-qualitative research–practice–policy bridge in health and social care systems reform.

As our practitioner co-authors note, the structure of music sessions is not rigidly pre-determined. Rather, they evolve based on community members’ feedback and what is observed during the activity. While some outcomes and goals be agreed upon in advance, the pathway towards achieving them is shaped by ongoing reflection and adaptation. In this way, the structure can remain cyclical and participant-informed, ensuring that co-creation is embedded throughout evaluation. Facilitators are encouraged to follow a format of Delivery, Observation, Reflection and Adjustment, where each session is built upon the previous one, all the while remaining responsive to emerging participant needs. 

It is important to emphasise the value of using the participants’ own words and reflections as main evidence of impact. In order to translate this into practice, there needs to be ongoing development of reflective tools, templates, or case studies to help future facilitators create music-based health-and-wellbeing programmes or initiatives that can link to different communities. The relational and emotional aspects of music (and other arts-informed) sessions are equally as important as ‘measurable’ outputs, so translation pieces should focus on capturing these dimensions as well as numerical data. In this way, the gap between supporting lived experience and meeting external demands for ‘measurable’ outcomes can be met. 

For researchers, practitioners and policymakers interested in forging a visionary path in Creative Health, we offer the following learning questions: How do we account for unexpected connections and contradictions between data sources? How can those relational aspects influence research for the better? How do lived and felt experiences shape the relationships between the people and objects in the research? How can we use rhizoanalysis to explore data in a flexible, non-linear way? (Masny, 2016). How can music sessions be designed to adapt to participant need, while still aligning with a project’s intended outcomes? Is the point even to align, or explore? How can facilitators remain open and responsive to feedback? How can evaluation frameworks be developed to meaningfully reflect felt experiences and the relational bonds that shape community engagement and wellbeing? And, crucially, how can facilitators, practitioners and policymakers balance the need for structure with the benefits of the unexpected and emergent activities? We invite these stakeholders to view flexibility not as a deviation from best practice, but as a vital component to it.

## Figures and Tables

**Table 1:** An assemblage of narratives summarised from four research projects/communities/practices.

Project Title	Sites	Participants	Context	Material Discursive Practices
Ah Cannae Sing	Community setting within in North Lanarkshire	Several community events including Gaelic edition: Hormones and Harmony; Power of the Female Voice	60–90 min workshops across the year	Workshops facilitated by professional musicians, usually by expert vocalists; developing materiality of voice, singing, body
All Ability Music	Cultural and community centres × 3	Weekly participation of 70 people (including support workers who also participate)	Sessions of 60 min	Combining percussion with songs in groups led by a professional musician
North Lanarkshire Community Choir	Cultural and community centres × 3	Weekly participation across three groups of 50 people	Sessions are 90 min	A non-auditioned choir led by professional musicians, open to anyone regardless of level or ability
Sensory Storytelling	Additional Support Needs (ASN) schools across North Lanarkshire And Mavisbank Schools	9 teachers and approximately 40 pupils	Blocks of 6–8 weeks in addition to one-off theatre events through Theatre in Schools Sessions last for 30 min	Sessions are facilitated by professional artists from drama and music sector with experience of delivering in ASN schools during the school timetable

## Data Availability

The data presented in this study are available on request from the corresponding author due to ethical reasons.

## Notes

1 Thing-power is a term introduced in ‘Vibrant Matter’ by Jane Bennett, who uses the phrase to capture the lively and active qualities of objects. She describes ‘things’ as having a powerful agency that act on us.

2 More-than-human refers to contexts in which multiple species and processes come together that the human world is necessarily embedded within, permeated by and indeed dependent upon (see Rodriguez-Garavito, 2024).

3 Material-discursive practices emphasise the inseparable entanglement of materiality and discourse in shaping reality. This means that material objects, actions and practices are not simply representations of discourse that are measurable, but actively participate in its creation and meaning-making.

4 ‘Sensing bodies’ is a term proposed by Cooke et al. (2023), which refers to the sensorial reverberations (audible, felt and observed) that spread, make and disrupt in bodies through intra-actions with materials and objects. The role of the sensing body, and the materiality of the body, is often under-theorized in comparison to knowing.

5 Jane Bennett (2010), a philosopher and political theorist, argues that objects and matter have ‘thing-power’, that is, the lively energy and/or resistant pressure that issues from one material assemblage and is received by others. The vitality of matter that we put to work here in this article is ‘making-with-music’ but it is also the focus on the material partnerships and meetings of bodies (human, non-human and more-than-human) that co-constitute practices.

6 Agential expressions express themselves as generative knowledges. Here we are inspired by Ursula Le Guin’s quotation: ‘We are volcanoes. When we [learners, teachers and researchers] offer our experience as our truth, as human truth, all the maps change. There are new mountains. That’s what I want—to hear you erupting.’ (Guin, 1989)

7 In spacetimemattering, “space, time, and matter are mutually constituted through the dynamics of iterative intra-activity. The spacetime manifold is iteratively reconfigured in terms of how material-discursive practices come to matter” (Barad, 2007, pp. 181–182).

## Abbreviations

The following abbreviations are used in this manuscript:

ASN	Additional Support Needs
